# The Risk of Dengue Virus Transmission in Dar es Salaam, Tanzania during an Epidemic Period of 2014

**DOI:** 10.1371/journal.pntd.0004313

**Published:** 2016-01-26

**Authors:** Leonard E. G. Mboera, Clement N. Mweya, Susan F. Rumisha, Patrick K. Tungu, Grades Stanley, Mariam R. Makange, Gerald Misinzo, Pasquale De Nardo, Francesco Vairo, Ndekya M. Oriyo

**Affiliations:** 1 National Institute for Medical Research, Dar es Salaam, Tanzania; 2 Department of Veterinary Microbiology and Parasitology, Sokoine University of Agriculture, Morogoro, Tanzania; 3 National Institute for Infectious Diseases, "L. Spallanzani", Rome, Italy; Colorado State University, UNITED STATES

## Abstract

**Background:**

In 2010, 2012, 2013 and 2014 dengue outbreaks have been reported in Dar es Salaam, Tanzania. However, there is no comprehensive data on the risk of transmission of dengue in the country. The objective of this study was to assess the risk of transmission of dengue in Dar es Salaam during the 2014 epidemic.

**Methodology/Principal Findings:**

This cross-sectional study was conducted in Dar es Salaam, Tanzania during the dengue outbreak of 2014. The study involved Ilala, Kinondoni and Temeke districts. Adult mosquitoes were collected using carbon dioxide-propane powered Mosquito Magnet Liberty Plus traps. In each household compound, water-holding containers were examined for mosquito larvae and pupae. Dengue virus infection of mosquitoes was determined using real-time reverse transcription polymerase chain reaction (qRT-PCR). Partial amplification and sequencing of dengue virus genome in infected mosquitoes was performed. A total of 1,000 adult mosquitoes were collected. Over half (59.9%) of the adult mosquitoes were collected in Kinondoni. *Aedes aegypti* accounted for 17.2% of the mosquitoes of which 90.6% were from Kinondoni. Of a total of 796 houses inspected, 38.3% had water-holding containers in their premises. Kinondoni had the largest proportion of water-holding containers (57.7%), followed by Temeke (31.4%) and Ilala (23.4%). The most common breeding containers for the Aedes mosquitoes were discarded plastic containers and tires. High Aedes infestation indices were observed for all districts and sites, with a house index of 18.1% in Ilala, 25.5% in Temeke and 35.3% in Kinondoni. The respective container indices were 77.4%, 65.2% and 80.2%. Of the reared larvae and pupae, 5,250 adult mosquitoes emerged, of which 61.9% were *Ae*. *aegypti*. Overall, 27 (8.18) of the 330 pools of *Ae*. *aegypti* were positive for dengue virus. On average, the overall maximum likelihood estimate (MLE) indicates pooled infection rate of 8.49 per 1,000 mosquitoes (95%CI = 5.72–12.16). There was no significant difference in pooled infection rates between the districts. Dengue viruses in the tested mosquitoes clustered into serotype 2 cosmopolitan genotype.

**Conclusions/Significance:**

*Ae*. *aegypti* is the main vector of dengue in Dar es Salaam and breeds mainly in medium size plastic containers and tires. The Aedes house indices were high, indicating that the three districts were at high risk of dengue transmission. The 2014 dengue outbreak was caused by Dengue virus serotype 2. The high mosquito larval and pupal indices in the area require intensification of vector surveillance along with source reduction and health education.

## Author Summary

Until 2010, little was known about Dengue in Tanzania. Since then, four outbreaks have been reported in Dar es Salaam City. This study was therefore carried out to assess the risk of transmission of dengue in Dar es Salaam during an outbreak in 2014. In this study adult mosquitoes were collected using carbon dioxide-propane powered traps. In addition, household compounds were visited and all water-holding containers examined for presence of mosquito larvae and pupae. Mosquito virus infection was determined using real-time reverse transcription polymerase chain reaction (qRT-PCR). Of the total of 1,000 adult mosquitoes collected, *Aedes aegypti* accounted for 17.2%. A total of 796 houses were inspected and 38.3% had water-holding containers in their premises. The most common breeding containers for the Aedes mosquitoes were discarded plastic containers and tires. High Aedes infestation indices were observed for all districts and sites, with a house and container indices ranging from 18.1–25.5% and 65.2–80.2%, respectively. The Breteaux indices were 30.6, 20.8 and 25.3 in Ilala, Kinondoni and Temeke, respectively. An overall 8.18% of mosquito pools were infected with dengue virus serotype 2. The overall maximum likelihood estimate of pooled infection rate of 8.49 per 1,000 mosquitoes was observed. This information is useful for the design of appropriate vector surveillance and control strategies in the City of Dar es Salaam.

## Introduction

Dengue is one of the most important mosquito-borne viral diseases in the tropics and subtropics. Although the exact global burden of dengue cases is unknown, recent estimates indicate that 390 million dengue infections occur annually. Of these, 96 million cases manifests clinically [1] with about half a million cases of dengue haemorrhagic fever requiring hospitalization [2]. Dengue is prevalent in Africa, though rarely reported. Statistics indicate that the disease has increased dramatically since 1980, with epidemics occurring in both eastern and western Africa [3–5]. The World Health Organization statistics indicate that 2.4% of the global dengue haemorrhagic fever cases occur in Africa and one-fifth of the population in the continent is at risk [6].

Although very little is known of the disease in Tanzania, dengue has an historical relationship with this East African country. The name dengue is believed to have been derived Kiswahili words “*ki denga pepo”*, meaning cramp-like seizure caused by an evil spirit. This condition was first described by Spanish sailors visiting the southern coast of Tanzania during the 15^th^ Century [4,7,8]. In the 1823 and 1870, dengue was reported on Zanzibar Islands [3]. Since then, three epidemiological surveys have indicated that different areas of Tanzania including Zanzibar, Iringa and Mbeya have reported varying prevalence of antibodies against dengue virus (DENV) in humans [9–11]. In 2010, 2012, 2013 and 2014, dengue outbreaks were reported in Dar es Salaam, with the worst outbreak in 2014 (Ministry of Health & Social Welfare, unpubl.). From January 2014 until end of May 2014, the cumulative number of confirmed and suspected dengue cases was 961 and 1,969, respectively (Ministry of Health and Social Welfare, unpubl.;http://promedmail.chip.org/pipermail/promed-eafr/2014-May/001515.html).

The most important vectors of dengue world-wide are *Aedes aegypti* and *Ae*. *albopictus*. Of the two mosquito species, *Ae*. *aegypti* is the most important vector because of being highly domesticated, strongly anthropophilic, a nervous feeder and a discordant species [12].

Despite the recent dengue outbreaks in Tanzania, there is no comprehensive data on the risk of transmission in the country. This study was therefore carried out to assess the risk of transmission of dengue in Dar es Salaam during an outbreak in 2014. Specifically, the study aimed to determine the (i) distribution and abundance of dengue vectors in Dar es Salaam; (ii) pattern of *Ae*. *aegypti* infestation and container productivity; and (iii) dengue virus infection in the mosquito vectors.

## Methods

### Study area

**Fig 1:**
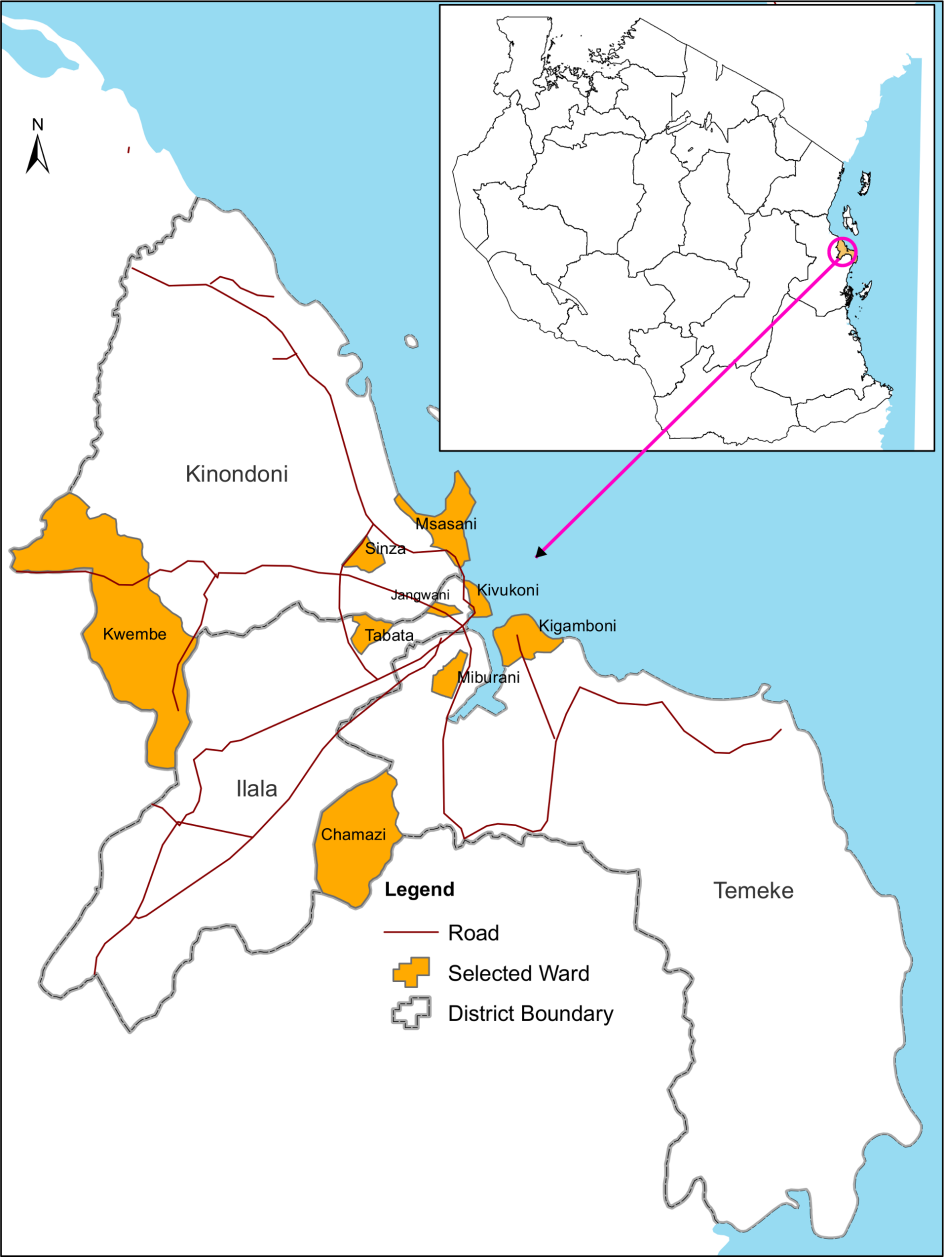
Study districts and sites in Dar es Salaam.

This study was carried out in Dar es Salaam region in eastern Tanzania and involved Kinondoni, Ilala and Temeke Districts (Fig 1). Dar es Salaam is the largest commercial City in Tanzania with an area of 1,339 km^2^. The region has a population of 4,364,541 (Ilala = 1,220,611; Kinondoni = 1,775,049; Temeke = 1,368,881), with 3,313 people per square kilometre and an annual growth rate of 5.6% [13]. The climate of Dar es Salaam is generally hot and humid with small seasonal and daily variations in temperature. The mean daily temperature is about 26°C, with the lowest and highest temperatures in July-August and February-March, respectively. Dar es Salaam has two dry and two rainy seasons. The main dry season lasts from June to September. The rainy seasons are from October-December and March-May. The total annual rainfall averages 1100 mm. Relative humidity is generally high, reaching 100% almost every night throughout the year, but falling to 60% during the day [14].

### Study design and sites

This cross-sectional study involved three sites (wards) in each district. The sites were selected based on the ecological and demographic characteristics. In Kinondoni the study sites were Msasani, Sinza and Kwembe. In Ilala, the sites were Kivukoni, Jangwani and Tabata. In Temeke the sites were Kigamboni, Miburani and Chamazi. Msasani, Kivukoni, and Kigamboni are high-income areas located along the Indian Ocean and characterized by sparsely populated neighbourhood. The sites have regular blocks with high standard dwellings, high vegetation coverage, piped water supply and regular garbage collection. The houses generally have large and shaded peridomestic environments. Sinza, Jangwani and Miburani are middle-class residential areas, with high density population and almost at the central location in the respective districts. The areas are characterized by poor garbage storage, collection and disposal. In these areas, shortage of piped water is common. Kwembe, Tabata and Chamazi are rural and located at the peripheral areas of the respective districts and characterized low population density.

The survey was conducted during the dengue outbreak in May-June 2014. A survey frame of households was obtained using a simple random sampling (without replacement) technique that was applied to select 100 households for the study in an identified block within each study site. A hand-held Geographical Positioning Systems (Magellan Meridian) was used to determine the geo-coordinates of the study sites.

### Adult mosquito collections

Adult mosquitoes were collected using carbon dioxide-propane powered Mosquito Magnet Liberty Plus traps (American Biophysics Corporation, Rhode Island, USA), fixed at nine sentinel sites (three sites in each district). Nine traps were set in each site and allowed to operate for three days consecutively. The traps were set in the morning and operated throughout the day and night. The mosquito catches were collected the following day and stored for further analysis.

### Larval and pupae surveys

Each household compound was examined for water holding containers and the presence of larvae and pupae of *Aedes* mosquitoes. Upon the entrance of the household premise, the yard and the interior of the household were thoroughly inspected for water-holding containers. All water-holding containers found were examined for presence of mosquito larvae and pupae. In each household compound, information on type of container, water type/state, approximate container volume, and water volume within the container and the number of larvae and pupae was recorded.

All larvae and pupae were collected using large-mouthed pipettes. In large containers a standard plastic dipper was used for larvae and pupae collection. Larvae and pupae were transported in lidded plastic containers and reared to adult stage in an insectary at Muhimbili University of Health Sciences. Pupae were kept in small water-filled plastic vials and placed into a netted cage until emergence of adults for accurate species identification. Adult mosquitoes were identified to genus or species using morphological identification keys [15]. The hatched mosquitoes were immediately killed by freezing and stored in liquid nitrogen for later screening of dengue virus.

### Dengue virus infection in mosquitoes

#### RNA extraction from mosquitoes

Pools of at least ten mosquitoes of the same species that emerged from larvae collections or adults collected by traps were ground before RNA was extracted using PureLink viral RNA/DNA kit (Invitrogen, Carlsbad, CA), following manufacturer’s instructions. Briefly, crushed mosquito suspensions were digested with 0.5 mg proteinase K at 56°C for 15 minutes in the presence of lysis buffer containing 5.6μg carrier RNA. Afterwards, proteins were precipitated by addition of absolute ethanol followed with pelleting by centrifugation. The lysate was passed through a viral spin column loaded with silica at 6800 g for 1 minute to trap RNA. RNA within the viral spin column was washed twice using a wash buffer before the column was dried by centrifugation at 13,000 g. RNA was eluted using 50μl of sterile RNase-free water and stored at -20°C until a qRT-PCR for dengue virus detection was performed.

#### Detection of dengue virus in mosquitoes

A qRT-PCR for the universal detection of the four dengue virus serotypes was performed using RealStar Dengue qRT-PCR kit version 1.0 (Altona Diagnostics GmbH, Hamburg, Germany), as previously described [16]. Briefly, RNA from mosquitoes was mixed with a master mix containing primers targeting amplification of all DENV serotypes 1–4. For each qRT-PCR run, a reaction containing DENV RNA supplied along with the kit and containing nuclease-free water were included as positive and negative controls, respectively. In addition, an internal control containing a heterologous amplification system to identify possible RT-PCR inhibition and to confirm the integrity of the reagents of the kit was included for each reaction during qRT-PCR. The qRT-PCR was performed by reverse transcription at 50°C for 10 min, denaturation at 95°C for 2 min followed by 45 cycles of denaturation at 95°C for 0.15 sec, annealing at 55°C for 0.45 sec and elongation at 72°C for 0.15 sec, and a final elongation step at 72°C for 7 min using an ABI 7500 Fast Real-Time PCR System (Applied Biosystems, Foster City, CA). Results were interpreted as strong positive for dengue virus RNA when the cycle threshold (CT) value was below 32, weakly positive when the CT value ranged between 33 and 38 and negative when CT value was above 39.

#### Dengue virus sequencing and phylogenetic analysis

A conventional RT-PCR for the amplification of the core-pre-membrane (CprM) region of dengue virus was performed using primers D1 and D2 [17]. All RT-PCR were run along with 1:10 dilutions of quantified (GCE/mL) laboratory-adapted, heat inactivated DENV1-4 strains (DENV-1 Hawaii 44, DENV-2 New Guinea C 44, DENV-3 H87 (Phillipines 56), DENV-4 H241 (Phillipines 241) as a positive control for DENV1-4 detection and nuclease-free water during RNA extraction and RT-PCR was used as the negative control. After amplification, RT-PCR products were sequenced directly using BigDye Terminator v3.1 Cycle Sequencing Kit followed by separation on a 3500 xL Genetic Analyser (Applied Biosystems, Foster City, CA).

The nucleotide sequences of dengue virus obtained in the present study were submitted to GenBank. A set of sequences representing dengue virus serotypes 1–4, together with the nucleotide sequence obtained in the present study, was used in phylogenetic analysis. Sequences were aligned using ClustalW algorithm and clustering pattern was determined by neighbour-joining method using Kimura-2 parameter option implemented in MEGA 6.06 [18].

### Data analysis

The data collected were entered in Epidata Version 3.1 software to develop a database which was later migrated to STATA (Stata Corps, 2007) for further analysis. Data were later cleaned and verified for quality by assessing a 10% of random selection of original forms that were compared with the entered data. From the database variables of interest that were calculated include the numbers of containers, infested containers, larvae and pupae collected. This was done for each type of container, and of infested containers found for every category of the variables measured. For populations larvae or pupae, three main methods were used to assess dengue transmission levels: (i) House index as the percentage of houses infested with larvae and / or pupae; (ii) Container index as the percentage of water-holding containers infested with larvae and/or pupae; and (iii) Breteaux index calculated as the number of positive containers per 100 houses inspected in a specific location. For adult mosquitoes, the proportion of Aedes mosquito out of those collected was assessed and all mosquitoes (including those hatched from pupae/larvae) were preserved in liquid nitrogen and later screened for dengue virus using qRT-PCR. Maps showing spatial variation of important indicators for dengue were produced. Infection rate was analysed using a PooledInfRate software version 4.0 to compute infection rates from pooled data [19].

### Ethical considerations

The study protocol was approved by the Tanzania Medical Research Coordinating Committee of the National Institute for Medical Research. Permission to conduct the study was sought from all administrative levels including regions, districts and wards. Verbal informed consent was obtained from the heads of households before the entrance and inspection of the house premises and/or installation of mosquito traps.

## Results

### Adult mosquito collections

A total of 1,000 adult mosquitoes were collected using Mosquito Magnet Liberty Plus traps. The largest proportion of adult mosquitoes (all species) was collected from Kinondoni (59.9%). *Ae*. *aegypti* accounted for 17.0% of the total adult mosquitoes collected; with the largest proportion (N = 154; 90.6%) been collected from Kinondoni (Table 1).

**Table 1:** Number (%) and species of adult mosquitoes collected by Mosquito Magnet Liberty Plus trap.

District	*Ae*. *aegypti*	*Ae*. *simpsoni*	*Culex quinquefasciatus*	*Anopheles gambiae*	*An*. *coustani*	Total
Ilala	10(7.3%)	0(0%)	127(92%)	1(0.7%)	0(0%)	138(13.9%)
Kinondoni	154(25.9%)	4(0.7%)	427(71.9%)	2(0.3%)	7(1.2%)	594(59.9%)
Temeke	6(2.2%)	0(0%)	259(96.6%)	1(0.2%)	2(1%)	268(27%)
Total	170(17%)	4(0.4%)	813(81.3%)	4(0.4%)	9(0.9%)	1,000

### Water-holding containers supporting mosquito breeding

**Fig 2:**
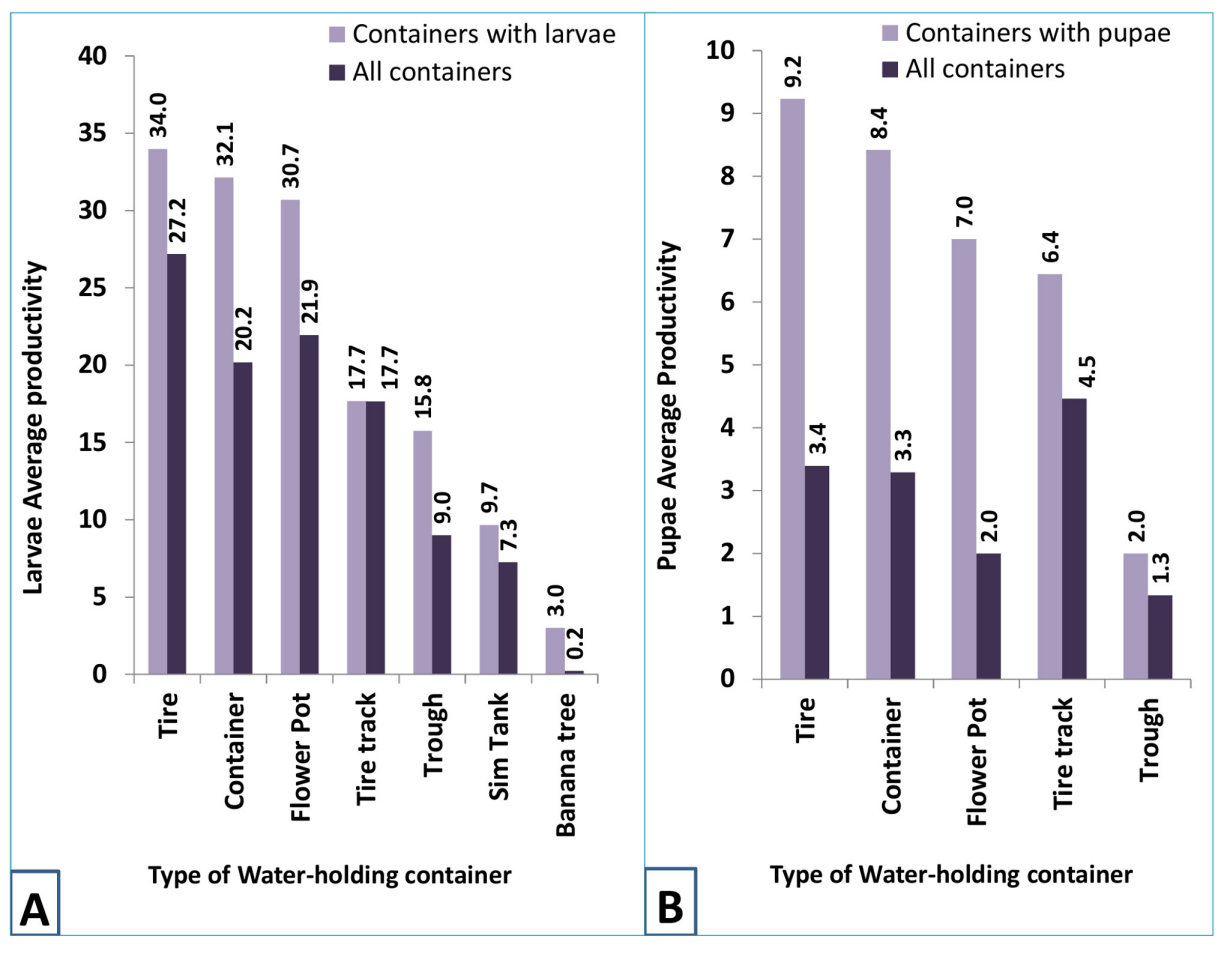
Average productivity of the mosquito larvae (A) and pupae (B) per type of water-holding container separating those found with larvae/pupae and all inspected.

A total of 796 houses were inspected for presence of potential Aedes mosquito breeding sites. Of these, 305 (38.3%) houses had water-holding containers in their premises. Kinondoni had the largest proportion (52.8%) of households with water-holding containers, followed by Temeke (29.8%) and Ilala (17.4%). A total of 219 (71.8%) of the households had at least one water-holding container with mosquito larvae and/or pupae. Water holding containers in 140 houses were found to harbour mosquito pupae. The most common breeding containers for the Aedes mosquitoes were medium-sized plastic containers and tires (Table 2). In terms of productivity, tires, plastic containers and flower pots were the most productive containers for both larvae and pupae in Dar es Salaam (Fig 2). Most of the flower pots (71.4%) were found in Kinondoni, followed by Temeke (21.4%) and Ilala (7.1%). Msasani and Sinza were the areas with the largest proportion of flower pots infested with larvae and/or pupae.

**Table 2:** Types of water holding containers infested with mosquito larvae/pupae by district and ward in Dar es Salaam.

Type of container	Ilala			Kinondoni			Temeke			
	Jangwani	Kivukoni	Tabata	Kwembe	Msasani	Sinza	Chamazi	Kigamboni	Miburani	All
Plastic containers (<5 litres)	28 (77.8%)	9 (28.1%)	10 (28.6%)	23 (31.9%)	21 (21.9%)	32 (31.1%)	15 (83.3%)	21 (44.7%)	37 (56.9%)	196 (38.9%)
Tire	4 (11.1%)	13 (40.6%)	15 (42.9%)	13 (18.1%)	26 (27.1%)	22 (21.4%)	0 (0%)	7 (14.9%)	15 (23.1%)	115 (22.8%)
Plastic containers (5–20litres)	2 (5.6%)	6 (18.8%)	6 (17.1%)	10 (13.9%)	14 (14.6%)	21 (20.4%)	0 (0%)	9 (19.2%)	1 (1.5%)	69 (13.7%)
Water tanks	1 (2.8%)	1 (3.1%)	3 (8.6%)	5 (6.9%)	1 (1%)	7 (6.8%)	0 (0%)	3 (6.4%)	2 (3.1%)	49 (4.6%)
Ceramic pots	1 (2.8%)	0 (0%)	0 (0%)	4 (5.6%)	5 (5.2%)	5 (4.9%)	1 (5.6%)	2 (4.3%)	2 (3.1%)	23 (4%)
Banana tree	0 (0%)	0 (0%)	1 (2.9%)	3 (4.2%)	5 (5.2%)	0 (0%)	1 (5.6%)	3 (6.4%)	0 (0%)	20 (2.6%)
Metal containers	0 (0%)	0 (0%)	0 (0%)	0 (0%)	9 (9.4%)	1 (1%)	0 (0%)	1 (2.1%)	0 (0%)	13 (2.2%)
Bottle	0 (0%)	0 (0%)	0 (0%)	1 (1.4%)	3 (3.1%)	3 (2.9%)	0 (0%)	1 (2.1%)	0 (0%)	11 (1.6%)
Others	0 (0%)	3 (9.4%)	0 (0%)	13 (18.1%)	12 (12.5%)	12 (11.7%)	1 (5.6%)	0 (0%)	8 (12.3%)	8 (9.7%)

The overall house larvae, container and Breteaux indices for Dar es Salaam were 27.5%, 71.8% and 8.2, respectively. House Index (HI) was highest in Kinondoni (35.3%) followed by Temeke (25.5%) and Ilala (18.1%). The Container Index (CI) was highest in Temeke (80.2%) followed by Ilala (77.4%) and Kinondoni (65.2%). This indicates that, though HI was higher in Kinondoni, the CI was higher in Temeke. Breteaux index was highest in Ilala (30.6), followed by Temeke (25.3) and was lowest in Kinondoni (20.8) (Table 3). Similarly, the highest pupal index was found in Temeke (52.9%), followed by Ilala (49.1%) and Kinondoni (41.0%). Analysis by site showed that Kivukoni had the highest CI while Sinza had the highest HI. The lowest HIs were observed in Chamazi, Jangwani, and Tabata. BIs were highest in Kivukoni and Tabata and lowest in Jangwani and Kwembe (Table 3).

**Table 3:** Number (%) of houses surveyed, water holding container (WHC), house larval and pupal and Breteaux indices by district and ward in Dar es Salaam.

District	Ward	No. (%) of houses surveyed	No. (%) of houses with WHC	No. of houses positive for Aedes larvae	Container index (%)	House larval index (%)	House pupal index (%)	Breteaux index
Ilala	Jangwani	107	22(20.6)	13	59.1	12.2	27.3	34.4
	Kivukoni	21	10 (47.6)	10	100	47.6	80.0	346.3
	Tabata	99	21 (21.2)	18	85.7	18.2	57.1	101.0
	***Subtotal***	***227 (28*.*5%)***	***53 (17*.*4)***	***41***	***77*.*4***	***18*.*1***	***49*.*1***	***30*.*6***
Kinondoni	Kwembe	94	48 (51.1)	24	50.0	25.5	20.8	42.26
	Msasani	86	52 (60.5)	32	61.5	37.2	38.5	66.4
	Sinza	99	61 (61.6)	49	78.7	49.5	59.0	82.4
	***Subtotal***	***297 (35*.*1%)***	***161 (52*.*8)***	***105***	***65*.*2***	***35*.*3***	***41*.*0***	***20*.*8***
Temeke	Chamazi	101	17 (16.8)	12	70.6	11.9	29.4	72.9
	Kigamboni	102	37 (36.3)	27	73.0	26.5	59.5	66.0
	Miburani	87	37 (42.5)	34	91.9	39.1	56.8	89.6
	***Subtotal***	***290 (36*.*4%)***	***91 (29*.*8)***	***73***	***80*.*2***	***25*.*2***	***52*.*8***	***25*.*3***
**Total**		**796**	**305 (38.3)**	**219**	**71.8**	**27.5**	**45.9**	**8.2**

### Dengue virus detection in mosquito pools

Of the hatched 5,250 adult mosquitoes, 3,250 *Ae*. *aegypti*, 800 *Ae*. *simpsoni* and 1,200 *Culex quinquefasciatus* were preserved in liquid nitrogen for later screening of dengue virus. A total of 368 mosquito pools, each containing up to 10 *Ae*. *aegypti* were processed to extract RNA. Of these, 330 pools were subjected to qRT-PCR for dengue virus detection. Overall, 27 (8.18) of the 330 pools of *Ae*. *aegypti* were positive for dengue virus. Of the 27 positive pools, 25 were from larval collections and two were from adult mosquito collected from Sinza. On average, the overall maximum likelihood estimate indicates pooled infection rate of 8.49 per 1,000 mosquitoes (95%CI = 5.72–12.16). There was no significant difference in pooled infection rates between the districts (Table 4). None of the mosquitoes collected in Kwembe and Chamazi was infected with dengue virus. Dengue viruses in the tested mosquitoes clustered into serotype 2 cosmopolitan genotype.

**Table 4:** Number of mosquito pools tested and DENV infection in *Aedes aegypti* by study sites in Dar es Salaam.

District	Site	Number of pools for RNA extraction	Number of pools tested for DENV	Number of positive pools	Percent of positive pools	Number of individuals (pooled)	Infection rate	Lower limit	Upper limit
Ilala	Jangwani	11	11	2	18.18	110	18.98	3.48	62.36
	Kivukoni	17	17	3	17.65	170	18.67	5.00	50.51
	Tabata	40	40	2	5.00	400	5.06	0.91	16.53
	***Subtotal***	***68***	***68***	***7***	***10*.*29***	680	10.73	4.76	21.13
Kinondoni	Kwembe	2	0	0	0.00	0	-	-	-
	Msasani	38	36	1	2.78	360	2.78	0.16	13.44
	Sinza	117	97	8	8.25	970	8.53	4.00	16.14
	***Subtotal***	***157***	***133***	***9***	***6*.*77***	1330	6.96	3.42	12.73
Temeke	Chamazi	39	36	0	0.00	360	0.00	0.00	0.00
	Kigamboni	64	54	2	3.70	540	3.73	0.67	12.21
	Miburani	40	39	9	23.08	390	25.56	12.66	46.71
	Subtotal	143	129	11	8.53	1290	8.84	4.68	15.32
	**Total**	**368**	**330**	**27**	**8.18**	**3300**	**8.49**	**5.72**	**12.16**

**Fig 3:**
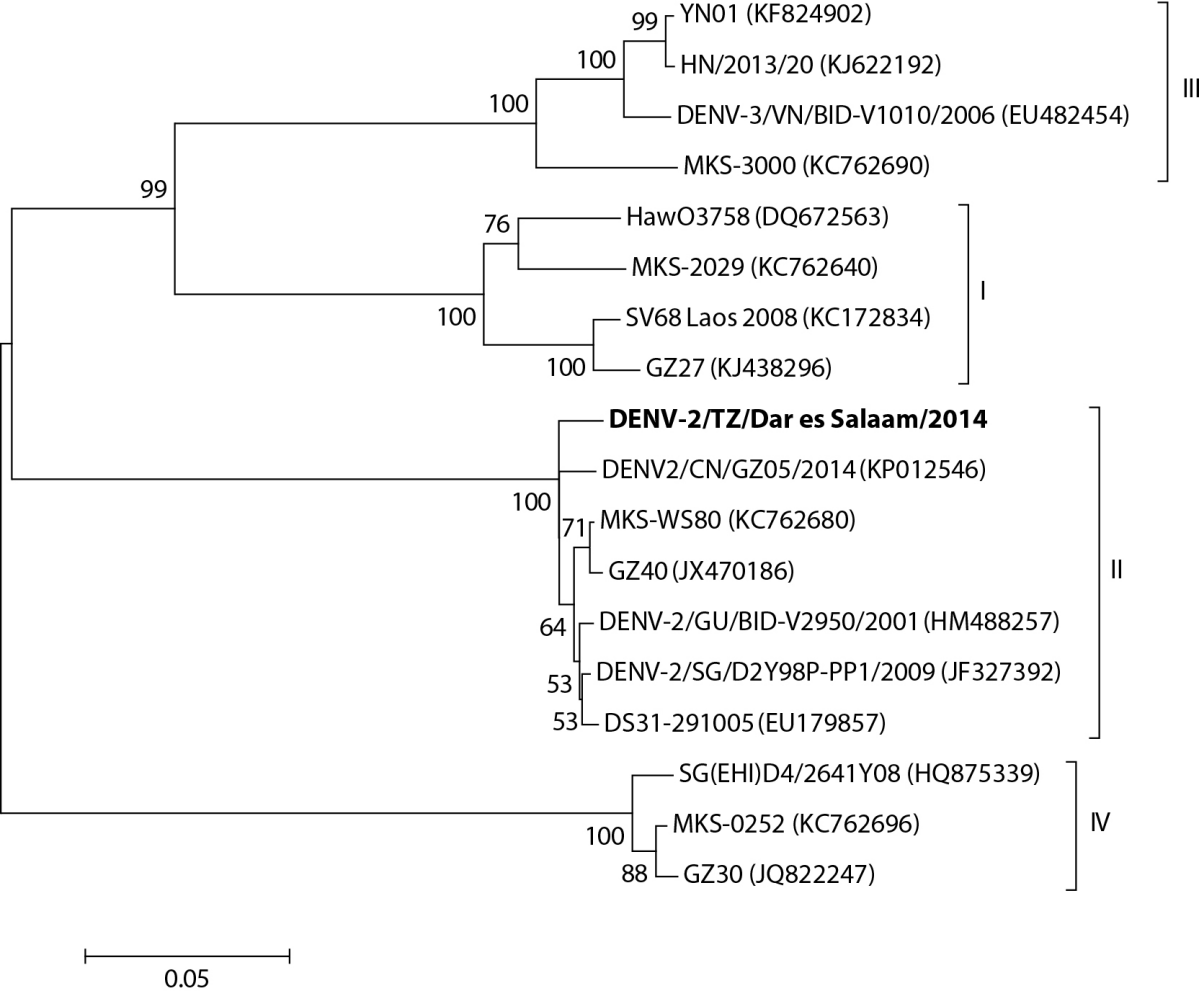
Neighbor-joining tree depicting the four serotypes of dengue virus. Dengue virus in mosquitoes collected in Dar es Salaam (indicated in bold) clustered into serotype 2. The percentage of replicate trees in which the associated taxa clustered together in the bootstrap test (1000 replicates) is shown adjacent to the nodes.

Partial nucleotide sequence of the dengue virus genome (CprM region) in mosquitoes collected were highly identical to and clustered with serotype 2 cosmopolitan genotype dengue viruses collected in Asia (Fig 3). These serotype 2 Asian dengue viruses were collected from human patients in 2007, 2008 and 2010 in Makassar (Indonesia), 2010 and 2014 in Guangdong (China) and 2005 and 2009 in Singapore.

## Discussion

In this study, *Aedes aegypti* accounted for about one fifth of the man-biting mosquitoes caught in Dar es Salaam. These findings provide the most up-date of data on *Ae*. *aegypti* in the region since 1980s. Over one-third of the inspected house premises had water-holding containers positive for larvae or pupae of Aedes mosquitoes. The most common breeding containers for *Ae*. *aegypti* in Dar es Salaam were discarded plastic containers and tires. Kinondoni district accounted for over half of the Aedes positive water holding containers. Most of these water holding containers are man-made, with only a few natural breeding sites identified. Previous studies in Tanzania have shown that in most places *Ae*. *aegypti* breeds in both artificial and natural sites [14,20, 21]. Similar to our findings, a study by Trpis [14] reported that tires, wrecked motor cars, water-pots, coconut shells, snail shells and tins were the most productive containers in some areas of the City during the early 1970s. It has already been shown that a part of the *Ae*. *aegypti* population in East Africa is maintained in some biotypes such as automobile dumps and coral rock holes by continuous breeding [14,20]. Discarded tires and animal watering pans have been reported to be the two most common larval breeding sites elsewhere [22].

*Ae*. *aegypti* has been reported as a common outdoor breeding mosquito even in rural areas of Tanzania. In the rural areas of Kilombero and Kilosa districts, Trpis [23] observed that more than a quarter of the water containers outside houses were harbouring *Ae*. *aegypti* larvae, while there was no breeding in containers indoors. In the same areas, clay pots, buckets, tins, drums used for storing water and tree holes were the common breeding sites. Similar to our findings, breeding of *Ae*. *aegypti* in banana flower bracts has been reported in southern-east Tanzania [23].

This study indicated that medium-sized containers and tires that are often susceptible to the rainfall regime are the most productive containers for *Ae*. *aegypti*. Similar findings have been observed in Trinidad [24] and Brazil [25]. In Brazil, the four most productive containers were found to generate up to 90% of total pupae. It has been reported that container productivity varies according to seasons and urbanization degree [25]. In this study container productivity varied by site and district and according to urbanization degree. The findings that the peri-urban areas of Dar es Salaam had low larval infestation levels suggest a low probability of dengue transmission in the areas. In a concurrent study the incidence of dengue was found to be relatively lower in the peri-urban areas ((F. Vairo et al., unpubl.).

The overall house and container index for Dar es Salaam was 27.5% and 71.8%, respectively. Previous studies elsewhere in Tanzania, have reported relatively lower larvae indices than those observed in our study [20]. In this current study, the higher Aedes mosquito HI, CI and BI were not surprising as the study was carried out during an epidemic that had already lasted for five months. The Breteaux indices in the three districts of Dar es Salaam (20.8–30.6%) were high. Higher Aedes indices, BI in particular, provides indication of geographical areas at high risk for dengue transmission [26]. Similar higher HIs have been reported in Singapore [27]. In Brazil, high infestation indices have been observed for both urban and sub-urban localities [28]. In a study in northern Ghana, similar higher HI, CI and BI have been reported in a recent study; with generally higher infestation rates during the dry season [29]. The spatial heterogeneities in *Ae*. *aegypti* larval density, HI, CI and BI found in this study have also been reported elsewhere in the world [30–34]. Identification of such heterogeneity is important in identifying areas for intervention.

The infection rates found in this study are relatively higher compared to many other reported elsewhere. The mosquito infection rates with dengue virus are of the order of 1% even in areas where transmission is ongoing [35–38]. Similar higher infection rates have been reported in a study in Merida, Mexico [39] and Odisha in India [40]. Although the mosquito infection rate in the current study was highest in Ilala followed by Temeke and Kinondoni, the incidence of dengue cases during the outbreak was highest in Kinondoni followed by Temeke and Ilala (Ministry of Health and Social Welfare, unpubl.). This is not surprising for the fact that, such a scenario is likely to be determined by dispersal of vector [37,41] which itself can vary over time [42], and is influenced by house density [43] and by human movement within and beyond the infection cluster [44]. In this study, the largest adult mosquito density per trap was observed in Kinondoni. Moreover, Human movements have been described to potentially confound dengue vector data that derive from residential areas alone as increasingly, evidence indicates that only a proportion of dengue infection are acquired in the individual’s own home, with the majority resulting from bites by virus-infected mosquitoes at their schools, workplaces or numerous locations far from the home [35,39,44,45]. In a recent study in rural Thailand [37], it was found that human and mosquito infections are positively associated with each other at small geographic and temporal scales.

Nucleotide sequencing and phylogenetic analysis of the CprM region of dengue virus found in mosquitoes in Dar es Salaam had highest identity and clustered with serotype 2 cosmopolitan genotype. Highest identity was with dengue serotype 2 strains collected from China, Indonesia and Singapore. These results indicate the widespread nature of dengue viruses and possible link to travel between Asia and Tanzania [46].

The recent outbreak of Dengue in Dar es Salaam occurred during the long rainy season (March-June, 2014). The high indices of *Ae*. *aegypti* in Dar es Salaam during the long rainy season were likely to be responsible for dengue outbreak. In a previous study of *Ae*. *aegypti* larval population in Dar es Salaam the density of the mosquito was high in April because of heavy rains and declined towards the end of June and remained at a very low level until the beginning of August [14]. This trend correlated with the decline of dengue cases observed in a parallel study (F. Vairo et al., unpubl.). However, a few studies have also shown dengue occurrence during the dry season. It is likely that in coastal areas of East Africa there are always some short scattered showers during the dry season that prevent these foci from completely drying out [20].

In conclusion, findings of this study indicate that the 2014 dengue outbreak in Dar es Salaam was caused by Dengue virus serotype 2 and most likely a result of the introduction from south-east Asia. *Ae*. *aegypti* is the only vector of Dengue in the region and breeds mostly in medium-sized plastic containers and tires. The high overall and site specific house indices and mosquito infection rates indicate that all three districts were at high risk of dengue transmission. With the high larval and pupal indices observed in the area there is need to intensify vector surveillance activities along with source reduction and public health education. The introduction of detailed systematic vector surveillance in Dar es Salaam and elsewhere in Tanzania, before, during, and after any dengue epidemic will offer an opportunity to analyse entomological information at different geographic units.
